# Source of Chronic Inflammation in Aging

**DOI:** 10.3389/fcvm.2018.00012

**Published:** 2018-02-22

**Authors:** Fumihiro Sanada, Yoshiaki Taniyama, Jun Muratsu, Rei Otsu, Hideo Shimizu, Hiromi Rakugi, Ryuichi Morishita

**Affiliations:** ^1^Department of Clinical Gene Therapy, Graduate School of Medicine, Osaka University, Suita, Japan; ^2^Department of Geriatric and General Medicine, Graduate School of Medicine, Osaka University, Suita, Japan

**Keywords:** hyper coagulation, cell senescence, inflammation, aging, IGFBP-5

## Abstract

Aging is a complex process that results from a combination of environmental, genetic, and epigenetic factors. A chronic pro-inflammatory status is a pervasive feature of aging. This chronic low-grade inflammation occurring in the absence of overt infection has been defined as “inflammaging” and represents a significant risk factor for morbidity and mortality in the elderly. The low-grade inflammation persists even after reversing pro-inflammatory stimuli such as LDL cholesterol and the renin–angiotensin system (RAS). Recently, several possible sources of chronic low-grade inflammation observed during aging and age-related diseases have been proposed. Cell senescence and dysregulation of innate immunity is one such mechanism by which persistent prolonged inflammation occurs even after the initial stimulus has been removed. Additionally, the coagulation factor that activates inflammatory signaling beyond its role in the coagulation system has been identified. This signal could be a new source of chronic inflammation and cell senescence. Here, we summarized the factors and cellular pathways/processes that are known to regulate low-grade persistent inflammation in aging and age-related disease.

## Introduction

At present, chronic inflammation is thought to be a risk factor for a broad range of age-related diseases such as hypertension, diabetes, atherosclerosis, and cancer. (1) Although age-related diseases may be partially preventable with lifestyle modifications, including diet, the burdens of unhealthy aging associated with lifestyle are increasing, both in developed and developing regions. Therefore, the elucidation of the sources and cellular pathways/processes of chronic inflammation is an urgent task. There are several possible factors that initiate and maintain a low-grade inflammatory response. These include aging, unbalanced diet, low level of sex hormones, and smoking. In contrast to young individuals, aged individuals have consistently elevated levels of inflammatory cytokines, especially interleukin-6 (IL-6) and tumor necrosis factor-α (TNF-α) (2), which may induce muscle atrophy and cancer through DNA damage. Visceral fat tissue from obese individuals can also produce both IL-6 and TNF-α, affecting systemic metabolism (3, 4). The accumulation of macrophages in visceral fat seems to be proportional to body mass index and appears to be a major source of low-grade persistent, systemic inflammation and insulin resistance in obese individuals (5, 6). Cigarette smoke contains multiple inducers of inflammation, especially reactive oxygen species. Indeed, chronic smoking increases production of several pro-inflammatory cytokines such as IL-6, TNF-α, and interleukin-1β (IL-1β) (7). Smoking also increases systemic inflammation and is an independent risk factor for several lifestyle-related diseases. Other inciting factors such as mental stress and periodontal disease have been reported. Importantly, the low-grade inflammation persists even after reversing the pro-inflammatory stimuli such as LDL cholesterol, the renin–angiotensin system (RAS), and smoking. These findings can be explained by the discovery of senescent associated secretory phenotype (SASP) and immunological imprinting.

**Figure 1:**
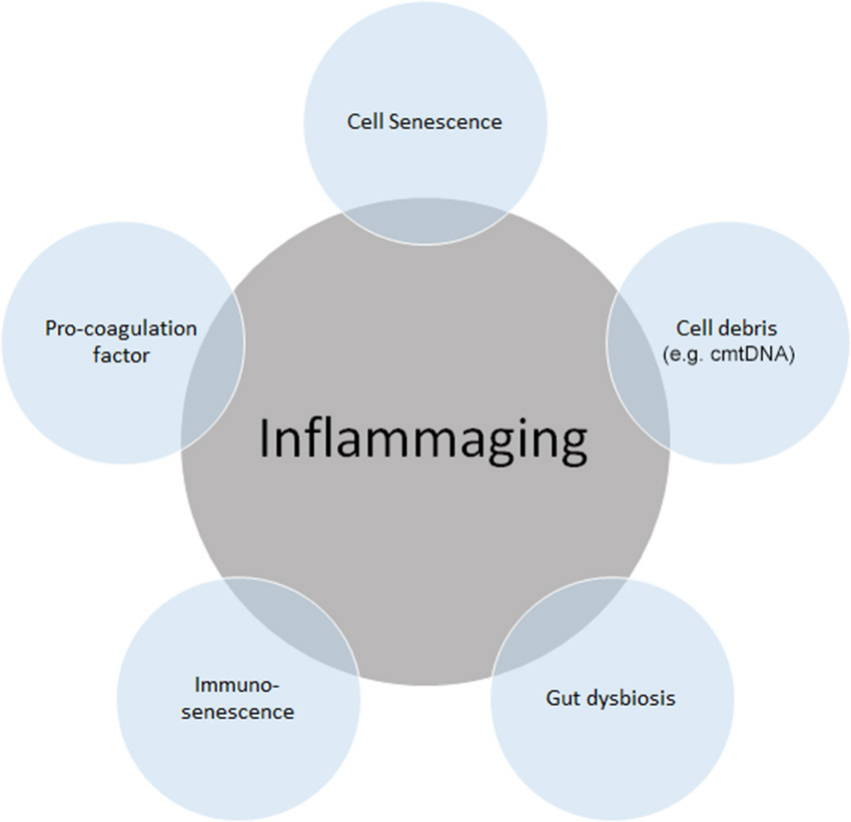
Source of “inflammaging”. Among the main causes of inflammaging, the accumulation of cell senescence, pro-coagulation factors, cell debris such as circulating mitochondrial DNA (cmtDNA), gut dysbiosis, and immune senescence is known to be the main causes of inflammaging. Inflammaging can also be influenced by many other factors, including age itself, reactive oxygen species (ROS), and those not directly related to inflammation, such as microRNAs (miRs) and agalactosylated N-glycans.

In this narrative review, we aimed to review the sources of chronic inflammation during aging (Figure 1). The cellular pathways/processes that are known to regulate the DNA damage response are also discussed.

## Source of Chronic Inflammation in Aging

Acute inflammation is indispensable for immune responses to invading pathogens or acute traumatic injuries. This process enables repair and cell turnover in multiple tissues. In contrast, chronic inflammation normally causes low-grade and persistent inflammation, leading to tissue degeneration. (8). Chronic, low-grade inflammation is a crucial contributor to various age-related pathologies and natural processes in aging tissue, including the nervous and the musculoskeletal system (9). Many tissues in the elderly are chronically inflamed, and inflammatory cytokines such as IL-6, IL-1β, and TNF-α are known to weaken the anabolic signaling cascade, including insulin and erythropoietin signaling, leading to the development of sarcopenia (10). The possible sources of chronic inflammation during aging, termed “inflammaging”, have been described previously (9).

### Cell Debris or Immunoglobulin Accumulation

Debris and immunoglobulin accumulation due to inappropriate cell elimination systems in aging trigger the innate immune system activation leading to persist inflammation. Glycosylation represents the most frequent post-translational modification of proteins. Protein-linked sugar chains play a variety of specific roles in the “fine-tuning” of interactions between cells and between molecules (11, 12). High-throughput analysis of the N-glycome, i.e., the sugar chains N-linked to asparagine, revealed new candidate biomarkers of natural aging, such as N-glycans devoid of galactose residues on their branches, in a variety of human studies (13–15) comparing healthy elderly people, centenarians, and their offspring, as well as in experimental animal models (16–18), such as the calorie-restricted mice model. These agalactosylated biantennary structures mainly decorate Asn297 of the Fc portion of IgG (IgG-G0) and are present in patients affected by progeria syndromes as well as a several autoimmune/inflammatory diseases. IgG-G0 exerts a pro-inflammatory effect through various mechanisms, including the lectin pathway of complement, binding to Fcγ receptors and formation of autoantibody aggregates. Similarly, the age-related accumulation of IgG-G0 activating the immune system can contribute to inflammaging. On the other hand, among the complex determinants of aging, mitochondrial dysfunction has attracted attention for some time. The consequences of age-related failing mitochondrial quality control include the release of mitochondria-derived damage-associated molecular patterns (DAMPs). Mitochondrial DAMPs, especially cell-free circulating mitochondrial DNA, have recently become the subject of intensive research because of their possible involvement in conditions associated with inflammation, such as aging and degenerative diseases (19, 20). Through their bacterial ancestry, these molecules contribute to increasing an inflammatory response by interacting with receptors similar to those involved in pathogen-associated responses.

### The Gut Mucosa and Microbiota of Elderly People

The barrier of the oral and gut mucosa against bacterial invasion deteriorates with age. Periodontal disease has also demonstrated to cause chronic low-grade inflammation (12). The gut microbiota of elderly people displays decreased diversity (21, 22). The abundance of anti-inflammatory microbiota, such as members of *Clostridium* cluster XIVa, *Bifidobacterium* spp., and *F. prausnitzii* are diminished in aged individuals (23). Toward R et al. demonstrated supportive data that the level of *Bifidobacterium* is inversely correlated with serum levels of inflammatory cytokines, such as TNF-α and IL-1β. Conversely, inflammatory and pathogenic microbiota, including *Streptococcus* spp., *Staphylococcus* spp., *Enterococcus* spp. and *Enterobacter* spp., are increased with age (23). Changes in the gut microbiota diversity in aged people might increase susceptibility to infectious agents by pathobionts colonization. Unique stool microbiota profiles were evident between healthy “community-dwelling elderly” and subjects with “residential long-term care” (24). These differences might be due to the disparate consumption of food in various groups, suggesting a new therapeutic target for prolonged healthy life years.

### Cell Senescence

Cellular senescence is defined as irreversible cell cycle arrest driven by a variety of mechanisms, including telomere shortening, genotoxic stress, mitogen stimuli, and inflammatory cytokines, that result in the activation of the p53 tumor suppressor and/or the cyclin-dependent kinase inhibitor p16 (25). It is evident that the number of senescent cells in several organs increases with age; these cells secrete multiple inflammatory cytokines, generating low-grade inflammation. This phenotype of senescent cells is termed the senescence-associated secretory phenotype or SASP, which recently has been proposed as the main origin of inflammaging in both aging and age-related diseases such as atherosclerosis, cancer, and diabetes (26–28). Increasing evidence has suggested that the clearance of senescent cells in animal models attenuates the progression of age-related disorders, including atherosclerosis and osteoarthritis (29–31). These data strongly support the hypothesis that senescent cell clearance, reprogramming of senescent cells, and the modulation of pro-inflammatory pathways related to the acquisition of SASP might be pursued as potential anti-aging strategies for combating age-related diseases and expanding the health span of humans.

### Immunosenescence

“Immunosenescence”, which is the age-related dysregulation of an innate immune system, is characterized by persistent inflammatory responses (32). Immunosenescence increases the susceptibility to malignancy, autoimmunity, and infections; decreases the response to vaccinations; and impairs wound healing (33, 34). Conversely, chronic inflammatory disease can accelerate the “immunosenescence” process. The mechanisms that underlie this persistent aging-associated basal inflammation remain incompletely understood but seem to involve changes in the numbers and functions of innate immune cells. Changes in the expression of pattern recognition receptors (PRRs), activation of PRRs by endogenous ligands associated with cellular damage, and unusual downstream signaling events of PRRs activation have been implicated to induce chronic cytokine secretion. Thus, together with cell senescence, dysregulation of immunological imprinting mediated by trained innate immunity might also contribute to persistent low-grade inflammation that occurs even after the initial stimulus has been removed.

### Coagulation and Fibrinolysis System

**Figure 2:**
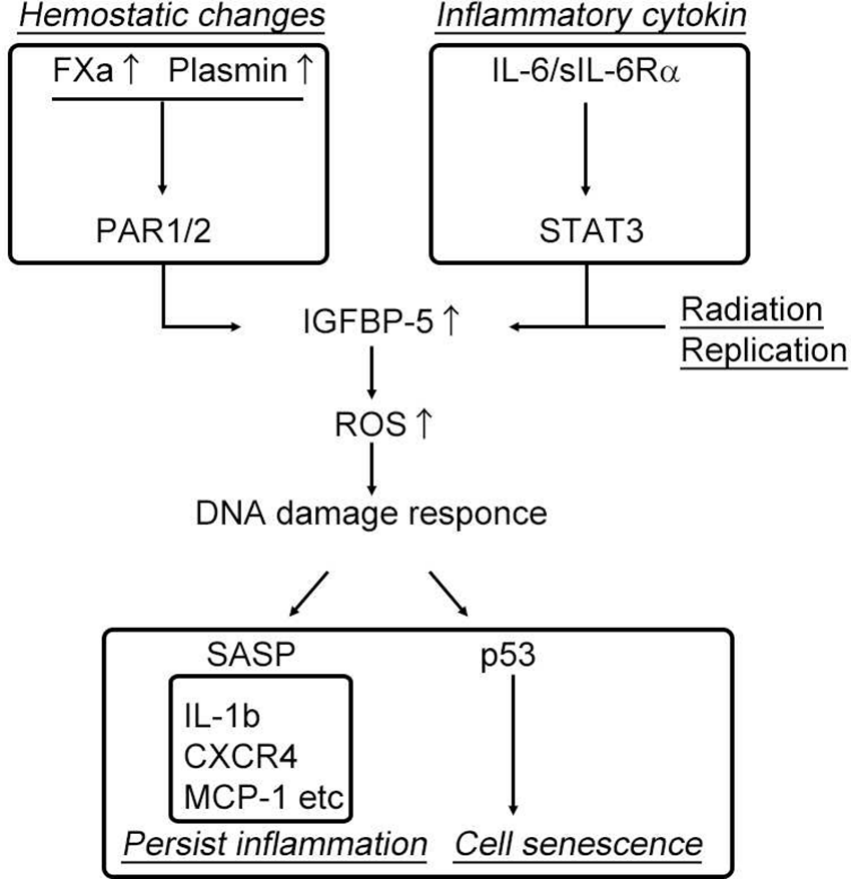
Activation of the coagulation cascade induces cell senescence and persistent inflammation.

Increased coagulation and fibrinolysis activity in the elderly has recently been implicated in enhanced inflammation through the protease-activated receptor, PAR (35–37) leading to age-related diseases such as atherosclerosis and lung fibrosis (38). The plasma concentrations of coagulation factor V, VII, VIII, and IX, have been reported to increase in healthy humans in conjunction with the physiological processes of aging (39, 40). In addition, fibrinogen (coagulation factor I) levels, a primary risk factor for thrombotic disorders proved in several clinical studies, has been shown to increase with advancing age. Additionally, we have recently identified that coagulation factor X is locally synthesized at high levels in human atherosclerotic plaques, specifically in endothelial cells, smooth muscle cells, and inflammatory cells (41). Thus, based on these observations, increased levels of plasma and local coagulation factors during physiological aging might account for the higher cardiovascular risk observed in the elderly. Additionally, a clinical trial, ATLAS ACS 2–TIMI, 51 investigators showed that the direct coagulation factor Xa inhibitor, rivaroxaban, reduced the risk of the composite endpoint of death from cardiovascular causes, myocardial infarction, and stroke in patients with a recent acute coronary syndrome event (42). Although the mechanism has not been elucidated, activation of the coagulation cascade following fibrinogen activation may increase thrombosis, and elevated levels of coagulation factor Xa and thrombin could enhance the inflammatory response through PAR-1/2, in addition to its roles in coagulation and the fibrinolysis system (43). Interestingly, PAR-1/2 signaling induced by coagulation factor Xa (FXa) and the fibrinolytic factor plasmin has been shown to increase insulin-like growth factor binding protein-5 (IGFBP-5) expression (37, 41, 44, 45), which plays decisive roles in cell senescence and inflammation. Kojima et al. demonstrated that IGFBP-5, a downstream mediator of signal transducer and activator of transcription 3 (STAT3), regulates interleukin-6 (IL-6)-induced reactive oxygen species production, subsequent DNA damage response, and senescence of fibroblast cells (46). As shown in the study by Yasuoka et al., IGFBP-5 induces a fibrotic phenotype by activating MAPK signaling and nuclear EGR-1 translocation that interacts with IGFBP-5 and promotes fibrotic and inflammatory gene transcription (47). Consistent with previous reports, our recent study demonstrated that FXa stimulation of smooth muscle cells, endothelial cells, and endothelial progenitor cells enhances cellular senescence through the early growth response-1 (EGR-1)-IGFBP-5-p53 pathway (37). These data imply that inflammaging, hyper-coagulability, and cell senescence might share a common pathway that is regulated by IGFBP-5 (Figure 2). Intriguingly, our recent experiment showed that the FXa- and IGFBP-5-positive areas were similarly distributed within human atherosclerotic plaques (41). These finding strongly suggest that locally produced coagulation factor Xa in atherosclerotic plaques might induce IGFBP-5 expression, enhancing cellular senescence with SASP, although the involvement of thrombin in this process is undeniable (48).

## Conclusions

Ideally, inflammation should subside immediately after elimination of the pathogen and insult to allow normal tissue to be rebuilt. However, low-grade persistent inflammation occurs in the majority of older people, leading to degeneration of several organs. There is strong evidence that the development of age-related multi-factorial conditions such as cancer, cardiovascular disease, Alzheimer’s disease, type II diabetes, frailty, sarcopenia, and osteoporosis is associated with low-grade elevations of circulating inflammatory mediators. Considering that aging is a complex process that results from a combination of environmental, genetic, and epigenetic factors, focusing future work on interventions addressing selectively destroying senescent cells, namely, “senolytic therapies” in the aging host rather than by treating symptoms of disease or attempting to block the effects of the multi-source of inflammaging, will offer improved therapeutic opportunities (49–52).

## Author Contributions

FS and YT performed experiment and organized manuscript. JM, HS, and RO performed experiments. HR and RM supervised this project.

## Conflict of Interest Statement

RM received research funding from Bayer Yakuhin, Ltd. Other authors have no conflicts of interests.
